# Supplementary material for: Patient preferences for incentives in Contingency Management interventions in methadone treatment: A best-worst scale analysis
Source: PLoS One. 2026 Jan 20;21(1):e0341359. doi: 10.1371/journal.pone.0341359 (PMC12818641; doi:10.1371/journal.pone.0341359)
Supplement: S1 Table — (DOCX) [file pone.0341359.s001.docx]

**S1 Table. Reasons for non-participation in the study (n=16)**

| **Reason for non-participation** | **n** |
| --- | --- |
| Work obligations (e.g., shift changes, being late for work) | 7 |
| Lack of time for any reasons other than work obligations | 3 |
| Household responsibilities (e.g., need to return home to care for a grandchild) | 2 |
| Emergency call during the interview | 1 |
| Others (specify: multiple study participants arrived at the same time; did not want to wait for their turn) | 3 |
