# Supplementary material for: Patient preferences for incentives in Contingency Management interventions in methadone treatment: A best-worst scale analysis
Source: PLoS One. 2026 Jan 20;21(1):e0341359. doi: 10.1371/journal.pone.0341359 (PMC12818641; doi:10.1371/journal.pone.0341359)
Supplement: Attachment — Submitted filename: Response to Reviewers_07Nov2025.docx [file pone.0341359.s004.docx]

November 7, 2025

Dear Reviewers,

We are pleased to resubmit our manuscript, “Patient Preferences for Incentives in Contingency Management Interventions in Methadone Treatment: A best-worst scale Analysis”. We greatly appreciate your thoughtful reviews and addressing your comments has strengthened our manuscript.

We have responded to the comment regarding potential selection bias; and have further elaborated on how the appealing incentives of the CM intervention could potentially improve adherence and retention in methadone treatment. We made changes to the manuscript of the tracked changes. Our detailed responses to the comment are provided on the table below.

Sincerely,

Authors

**Reviewer 1**

| **Comment** | **Response** |
| --- | --- |
| 1. This is a well-written report on an innovative study that has potential to be helpful to clinicians, policy-makers, and researchers interested in implementing contingency management (CM) in low-resourced methadone clinics for opioid use disorder. I have been active in CM research for many years and was unfamiliar with the interesting best-worst analysis method used in this study. I enjoyed learning about it and suspect others may as well. I commend the authors on this innovative study and well-crafted report. I have no recommendations for changes. | We thank the reviewer for the review and support. |

**Reviewer 2**

| **Comment** | **Response** |
| --- | --- |
| 1. The study aimed to characterize and measure patient preferences for low-cost incentives within a Contingency Management (CM) framework designed to enhance adherence and retention in Methadone Maintenance Treatment (MMT). It specifically addresses the challenge of implementing CM in settings with limited resources, like a low to medium income country as Vietnam. This study provides empirically-driven insights into patient preferences for low cost incentives. However, there are potential selection bias that need to be discussed. While the authors mentioned they prioritized approaching participants with poor adherence or a history of discontinuation, they did not show if this subgroup behave significantly different from a more general population. | We thank the reviewer for the insightful comment.  As we conducted this study to explore patients’ preferred incentives to reinforce retention and adherence, we prioritized choosing participants with this target behavior. We clarified this point in “Participants” subsection (page 7) and as follows:  **“Although poor adherence or previous discontinuation of methadone was not an explicit inclusion criterion, the target population we aimed to reach out consisted of patients currently receiving methadone who demonstrated poor adherence and/or were at high risk of treatment discontinuation. These aspects were target behaviors for the CM interventions. We worked closely with clinical staff to identify and approach this population as they presented in the study sites.”**  In this target population, we anticipate a minimal impact of selection bias with less than 10% of non-participation (S1 Table).  However, the selection of patients with poor adherence might limit our generalizability to all patients in methadone, thus, we have a few sentences in the last paragraph of Discussion to talk about potential generalizability. |
| 2. Also, as a cross-sectional study, it measured stated preference, which may not be directly translated into actual treatment efficacy. It is not clear, if the preferred incentives would improve adherence and retention in the long run, which further evidence should be presented by the authors or reference materials. | We thank the reviewer for the important point. We added some sentences in Introduction (please refer to Page 5) for more elaboration on this point. Also, please see below our brief explanation.  As we laid out in Introduction, evidence indicated that the effectiveness of CM on treatment adherence and retention have been seen in both high and low/middle income countries [18,21,22]. But the implementation of CM in low/middle income countries faces particular challenges (which can hinder the CM’s efficacy) [21]. That said, we need to explore ways to strengthen the implementation of CM to ensure its effectiveness in low-income, real-world settings. To improve the implementation, some upstream implementation outcomes, such as acceptability and feasibility, were particularly appropriate to focus on at the beginning. Thus, understanding patients’ preferences has the potential to enhance acceptability and implementation of CM intervention, which ultimately ensures the effectiveness of CM (this effectiveness has been shown in previous studies, some examples as reference 18, 21 and 22).  But we totally agree with the reviewer that in the long run, further research with a focus on the relationship between these types of incentives and longer-term retention and adherence in methadone treatment are needed. |
