# Supplementary material for: Patient preferences for incentives in Contingency Management interventions in methadone treatment: A best-worst scale analysis
Source: PLoS One. 2026 Jan 20;21(1):e0341359. doi: 10.1371/journal.pone.0341359 (PMC12818641; doi:10.1371/journal.pone.0341359)
Supplement: Attachment — Submitted filename: Response to Reviewers_22Dec2025.docx [file pone.0341359.s005.docx]

December 22, 2025

Dear Editor and Reviewers,

We greatly appreciate your clarification and comments to help improve our manuscript. We are pleased to resubmit our manuscript, “Patient Preferences for Incentives in Contingency Management Interventions in Methadone Treatment: A best-worst scale Analysis”.

Please see below our responses. We also made changes in the resubmitted manuscript accordingly.

Sincerely,

Authors

| **Comment** | **Response** |
| --- | --- |
| Please consider the following:   1. fully describe the inclusion and exclusion criteria. If staff were directed to 'select' or 'approach' people based on un-documented inclusion or exclusion criteria, this needs to be further elaborated on. This kind of subjective selection can have significant impacts on your results, interpretations, and conclusions.  Please address this more fully with information about who did the approach for the study and how they decided if someone was eligible. Please state in the Results how many people were approached to participate, and also, please state how many and the proportion of those approached who agreed to participate. | We thank the editor for the clarification and comment. We elaborated on the process of selection in participants subsection (Page 8-9)*, then discussed further on the risk of selection bias in Discussion (Page 23-24).  We clarified in Results (Page 14) regarding the number of patients who were approached. As they were first verbally approached by clinical staff, then patients who were interested in the study were referred to research assistants to do formal eligibility screening, introduction and informed consent. We did not document the total number of patients verbally approached by clinical staff; but we documented the number of patients after they met with research assistants, who declined to participate. |
| 2. Please state the dates during which the study was conducted. | We thank the editor for requesting more information. We added time period for data collection in the study design section (Page 6) which was from Nov 14 to 26, 2023 with one day spent collecting data at one site (i.e., 6 days for 6 sites in total). |
| 3. Please add information about who consented the participants, and was it written? Was participation anonymous, or were names collected? | We thank the editor for this comment. We added a paragraph on the procedure when informed consent form was obtained (Page 11). We added the confidentiality aspect in Page 9 where we mentioned IRB approval. |
| 4. ABSTRACT: your conclusion paragraph seems to merely reiterate the results.  Can you elaborate more on how these results might inform implementation? | We thank the editor for the comment. We revised and clarified the abstract conclusion. We suggest it would be beneficial for the future contingency management (CM) intervention to consider or start with the most preferred incentives as their first-line rewards to offer in the CM interventions. |

*all pages are referred to the “Revised manuscript with track changes” version.
